# Stable and non-toxic ultrasmall gadolinium oxide nanoparticle colloids (coating material = polyacrylic acid) as high-performance *T*_1_ magnetic resonance imaging contrast agents

**DOI:** 10.1039/c7ra11830a

**Published:** 2018-01-16

**Authors:** Xu Miao, Son Long Ho, Tirusew Tegafaw, Hyunsil Cha, Yongmin Chang, In Taek Oh, Ahmad Mohammad Yaseen, Shanti Marasini, Adibehalsadat Ghazanfari, Huan Yue, Kwon Seok Chae, Gang Ho Lee

**Affiliations:** Department of Chemistry, Department of Nanoscience and Nanotechnology (DNN), College of Natural Sciences, Kyungpook National University (KNU) Taegu 41566 South Korea ghlee@mail.knu.ac.kr; Department of Molecular Medicine and Medical & Biological Engineering, DNN, School of Medicine, KNU and Hospital Taegu 41566 South Korea ychang@knu.ac.kr; Department of Biology Education, DNN, Teachers' College, KNU Taegu 41566 South Korea

## Abstract

For use as positive (*T*_1_) magnetic resonance imaging contrast agents (MRI-CAs), gadolinium oxide (Gd_2_O_3_) nanoparticle colloids (*i.e.* nanoparticles coated with hydrophilic ligands) should be stable, non-toxic, and ultrasmall in particle diameter for renal excretion. In addition, they should have a high longitudinal water proton relaxivity (*r*_1_) and *r*_2_/*r*_1_ ratio that is close to one (*r*_2_ = transverse water proton relaxivity) for high-performance. In this study, we report ultrasmall Gd_2_O_3_ nanoparticle colloids [coating material = polyacrylic acid, *M*_w_ = ∼5100 Da] satisfying these conditions. The particle diameter was monodisperse with an average value of 2.0 ± 0.1 nm. The colloidal suspension exhibited a high *r*_1_ value of 31.0 ± 0.1 s^−1^ mM^−1^ and *r*_2_/*r*_1_ ratio of 1.2, where *r*_1_ was ∼8 times higher than that of commercial Gd-chelates: the cooperative induction model was proposed to explain this. The effectiveness of the colloidal suspension as a high-performance *T*_1_ MRI-CA was confirmed by taking *in vivo T*_1_ MR images in a mouse after intravenous administration. Highly positive contrast enhancements were observed in various organs of the mouse such as the liver, kidneys, and bladder. The colloidal suspension was then excreted through the bladder.

## Abstract 2

Stable and non-toxic ultrasmall Gd_2_O_3_ nanoparticle colloids as high-performance *T*_1_ MRI-CA were developed.

## Graphical abstract

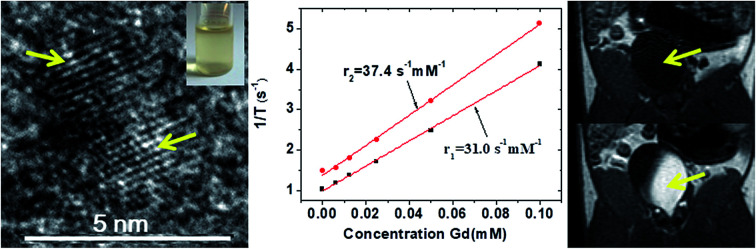

## Introduction

Magnetic resonance imaging contrast agents (MRI-CAs) allow us to discriminate between normal and abnormal tissues inside the body through the differential contrast enhancement between them.^1–3^ This occurs owing to the differential population of the MRI-CAs between them. Because the proton spin relaxation times are shortened by the MRI-CAs, higher contrast MR images can be observed in regions where the MRI-CAs are more concentrated. The currently used positive (*T*_1_) MRI-CAs are molecular Gd-chelates and generally exhibit longitudinal water proton relaxivity (*r*_1_) values of 3–5 s^−1^ mM^−1^ (*r*_2_/*r*_1_ = 1.1–1.2), where *r*_2_ is the transverse water proton relaxivity.^4–6^ However, gadolinium oxide (Gd_2_O_3_) nanoparticles have exhibited *r*_1_ values higher than these values.^7–9^ Therefore, the synthesis of high-performance (or powerful) *T*_1_ MRI-CAs using Gd_2_O_3_ nanoparticles^7–9^ including various Gd-containing polymeric^5^ and nanosystems^10–13^ is currently a hot topic.

To be applied as *T*_1_ MRI-CAs, Gd_2_O_3_ nanoparticle colloids (*i.e.* nanoparticles coated with hydrophilic ligands) should be stable, non-toxic, and ultrasmall in particle diameter for renal excretion. In addition, they should have a high *r*_1_ value and *r*_2_/*r*_1_ ratio that is close to one for high-performance. For colloidal stability and biocompatibility, Gd_2_O_3_ nanoparticles should be coated with hydrophilic and biocompatible ligands. Here, a more hydrophilic ligand is preferred for surface-coating because it can afford a higher colloidal stability and, importantly, a higher *r*_1_ value as well,^14–16^ because it can allow more water molecules to access the nanoparticle. Renal excretion of Gd_2_O_3_ nanoparticle colloids is essential for *in vivo* applications because the Gd^3+^ ion is toxic. Gd^3+^ ions can cause nephrogenic systemic fibrosis (NSF) when released inside the body.^17–19^ Therefore, the particle diameter should be less than 3 nm for renal excretion.^20–22^ In a previous study, *r*_1_ was optimal at a particle diameter of ∼2 nm.^23^ This implies that high performance *T*_1_ MRI-CAs can be synthesized using ultrasmall Gd_2_O_3_ nanoparticle colloids with a particle diameter of ∼2 nm, as thus investigated in this study. In addition this study as a continuation of the previous study^23^ employed polyacrylic acid (PAA) as surface-coating ligand to obtain stable nanoparticle colloids and as a result to achieve high-performance *in vivo T*_1_ MRI.

Several Gd-nanosystems with very high *r*_1_ values have been reported.^14,24–26^ Ultrasmall Gd_2_O_3_ nanoplates with a diameter of 2 nm coated with PAA–octylamine (OA) showed an *r*_1_ value of 47.2 s^−1^ mM^−1^ (*r*_2_/*r*_1_ = 1.7) at 1.41 T.^14^ Dense Gd^3+^ ions conjugated on the surface of carbon nanotubes (CNTs) exhibited an *r*_1_ value of 70 s^−1^ mM^−1^ (*r*_2_/*r*_1_ = 1.5) at 1.41 T and 37 °C (ref. 24) and those prepared inside CNTs showed an *r*_1_ value of 94 s^−1^ mM^−1^ at 1.41 T and 37 °C.^25^ Gd^3+^-ion clusters within ultra-short single-walled CNTs exhibited an *r*_1_ value of 164 s^−1^ mM^−1^ in an 1% sodium dodecyl benzene sulfate aqueous solution and 173 s^−1^ mM^−1^ in a pluronic F98 surfactant aqueous solution at 1.41 T and 40 °C.^26^ These *r*_1_ values are significantly higher than those of Gd-chelates.^4–6^ To understand these high *r*_1_ values including that observed in this study, a cooperative induction model was proposed. In this model, several Gd^3+^ ions exposed on the nanoparticle surface or in the Gd^3+^ ion cluster cooperatively induce the longitudinal water proton relaxation of a water molecule. Using this model, we gave a successful explanation for these high *r*_1_ values. It is worth noting that for *in vivo* applications, spherical nanoparticles are preferred over other shapes and complex systems because of their better transport through capillary vessels. In this respect, the ultrasmall Gd_2_O_3_ nanoparticle colloid is one of the potential high-performance *T*_1_ MRI-CAs.

**Fig. 1:**
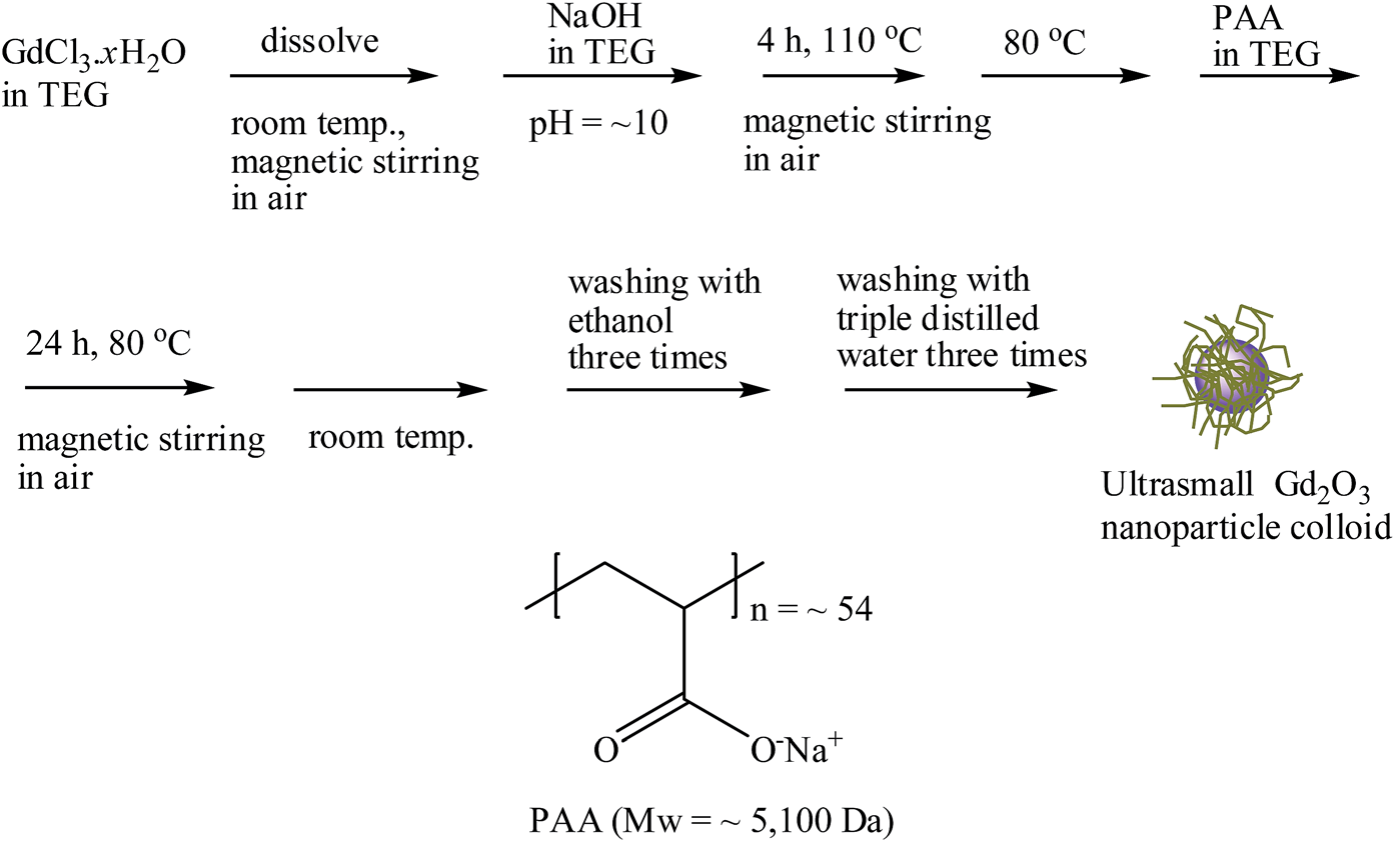
Reaction scheme for the one-pot synthesis of ultrasmall Gd_2_O_3_ nanoparticle colloid and PAA structure.

In this study, we report the facile one-pot synthesis and *in vitro* and *in vivo* characterization of ultrasmall Gd_2_O_3_ nanoparticle colloids (core *d*_avg_ = 2.0 nm; coating material = PAA, *M*_w_ = ∼5100 Da). PAA was directly coated on the nanoparticle surface. PAA is a well-known biocompatible and hydrophilic polymer,^27^ possessing one carboxyl group per monomer unit (as a result, numerous carboxyl groups per polymer) (Fig. 1). PAA binds to the nanoparticle surface through electrostatic bonding between its carboxyl groups and Gd^3+^ ions exposed on the nanoparticle surface. Multiple binding of PAA through its many carboxyl groups with a nanoparticle allows strong binding with the nanoparticle, leading to excellent colloidal stability and biocompatibility. To demonstrate that ultrasmall Gd_2_O_3_ nanoparticle colloids can be used as a high-performance *T*_1_ MRI-CA, we measured the cellular toxicity, water proton relaxivities, and *in vivo T*_1_ MR images after intravenous administration. In addition, the cooperative induction model was employed to explain the observed high *r*_1_ value.

## Experimental

### Chemicals

All chemicals including GdCl_3_·*x*H_2_O (99.9%), NaOH (>99.9%), triethylene glycol (TEG) (99%), and PAA (*M*_w_ = ∼5100 Da) were purchased from Sigma-Aldrich and used as received. Ethanol (99%, Duksan, South Korea) was used for initial nanoparticle washing. Triple distilled water was used for final nanoparticle washing and the preparation of an aqueous colloidal suspension.

### Synthesis of ultrasmall Gd_2_O_3_ nanoparticle colloids

The one-pot synthesis of ultrasmall Gd_2_O_3_ nanoparticle colloids (coating material = PAA) is shown in Fig. 1. Three solutions were prepared: (1) a precursor solution made of 1 mmol of GdCl_3_·*x*H_2_O and 10 mL of TEG in a 100 mL three-neck-flask, (2) a solution made of 4 mmol of NaOH and 10 mL of TEG in a 100 mL beaker, and (3) 0.05 mmol of PAA in a mixture of 5 mL of TEG and 5 mL of triple distilled water in a 100 mL beaker. Solution (1) was magnetically stirred at room temperature under atmospheric conditions until the precursor was dissolved in TEG. Solution (2) was slowly added to the precursor solution until the pH of the solution reached ∼10. The resulting mixture solution was gradually heated to 110 °C and magnetically stirred for 4 h. The reaction temperature decreased to 80 °C. Solution (3) was slowly added to the reaction solution, and then the reaction solution was magnetically stirred for 24 h at that temperature. The product solution was cooled to room temperature and transferred to a 500 mL beaker. Then, 400 mL of ethanol was added to the product solution, which was magnetically stirred for 10 min. The product nanoparticle colloids were allowed to settle to the bottom of the beaker for a week in a refrigerator (4 °C). The transparent supernatant was decanted to remove unreacted precursors, free PAA, and TEG. This washing process with ethanol was repeated three times. To remove ethanol, the product solution was diluted with 400 mL of triple distilled water and then concentrated using a rotary evaporator. This process was repeated three times. The obtained concentrated colloidal suspension was split into two equal parts: one part was diluted with triple distilled water to prepare a colloidal suspension (∼30 mM Gd), and the other part was dried in air to obtain a powder sample for various characterizations.

### General characterizations of ultrasmall Gd_2_O_3_ nanoparticle colloids

The particle diameter (*d*) of the colloidal suspension was measured using a high-resolution transmission electron microscope (HRTEM) (Titan G2 ChemiSTEM CS Probe, FEI) operated at 200 kV. A drop of the sample solution diluted in ethanol was placed on a carbon film supported by a 200-mesh copper grid using a micropipette (Eppendorf, 2–20 μL) and allowed to dry in air at room temperature. The copper grid with nanoparticle colloids was subsequently placed inside the HRTEM vacuum chamber for measurement.

The Gd-concentration of the colloidal suspension was determined using an inductively coupled plasma atomic emission spectrometer (ICPAES) (IRIS/AP, Thermo Jarrell Ash Co.). All samples were pre-treated with acids to completely dissolve the nanoparticle colloids in solution before measurement.

A dynamic light scattering (DLS) particle size analyzer (UPA-150, Microtrac) was used to measure the hydrodynamic diameter (*a*) of the nanoparticle colloids using a sample solution (∼0.01 mM Gd).

The colloidal stability was investigated by measuring the backscattering (BST) of near infrared (NIR) beam (880 nm) as a function of height (*h*) (*h* = 5 to 10 mm from the vial bottom containing the sample solution) and time (*t*) for *t* = 0 to 3 days using a Turbiscan (Turbiscan AGS, Formulaction).

A multi-purpose X-ray diffractometer (X'PERT PRO MRD, Philips) with unfiltered CuKα radiation (*λ* = 0.154184 nm) was used to characterize the crystal structures of the powder samples. A scan step of 2*θ* = 0.03° and a scan range of 2*θ* = 15–100° were used.

The attachment of PAA to ultrasmall Gd_2_O_3_ nanoparticles was probed by recording the Fourier transform infrared (FT-IR) absorption spectra using an FT-IR absorption spectrometer (Galaxy 7020A, Mattson Instruments, Inc.) and employing powder samples pelletized with KBr. The scan range was 400–4000 cm^−1^.

A thermogravimetric analysis (TGA) instrument (SDT-Q600, TA Instruments) was used to estimate the surface-coating amount by recording the TGA curve between room temperature and 900 °C under an air flow. An average amount of surface-coated PAA was estimated from the mass loss, after taking into account the water and air desorption between room temperature and ∼105 °C. The amount of Gd_2_O_3_ was estimated from the remaining mass. It was also estimated by measuring the Gd weight percent of a powder sample using an ICPAES. After TGA, the remaining sample was collected and subjected to X-ray diffraction (XRD) analysis.

### Relaxivity and map image measurements

The longitudinal (*T*_1_) and transverse (*T*_2_) relaxation times and the longitudinal (*R*_1_) and transverse (*R*_2_) map images were measured using a 1.5 T MRI scanner (GE 1.5 T Signa Advantage, GE Medical Systems) equipped with a knee coil (EXTREM). Aqueous dilute solutions (0.1, 0.05, 0.025, 0.0125, and 0.00625 mM Gd) were prepared *via* dilution of the concentrated colloidal suspension with triple distilled water. These dilute solutions and triple distilled water were then used to measure the *T*_1_ and *T*_2_ relaxation times and *R*_1_ and *R*_2_ map images. Subsequently, the *r*_1_ and *r*_2_ values of the colloidal suspension were estimated from the slopes of the plots of 1/*T*_1_ and 1/*T*_2_, respectively, *versus* the Gd concentration. *T*_1_ relaxation time measurements were performed using an inversion recovery method. In this method, the inversion time (TI) was varied at 1.5 T, and the MR images were acquired at 35 different TI values in the range from 50 to 1750 ms. The *T*_1_ relaxation times were then obtained from the nonlinear least-squares fits to the measured signal intensities at various TI values. For the measurements of *T*_2_ relaxation time, the Carr–Purcell–Meiboom–Gill pulse sequence was used for multiple spin-echo measurements. Then, 34 images were acquired at 34 different echo time (TE) values in the range from 10 to 1900 ms. The *T*_2_ relaxation times were obtained from the nonlinear least-squares fits to the mean pixel values for the multiple spin-echo measurements at various TE values. The parameters used for the measurements were as follows: external MR field (*H*) = 1.5 T; temperature (*T*) = 22 °C; number of acquisitions (NEX) = 1; field of view (FOV) = 16 cm; FOV phase = 0.5; matrix size = 256 × 128; slice thickness = 5 mm; pixel spacing = 0.625 mm; pixel band width = 122.10 Hz; and repetition time (TR) = 2000 ms.

### *In vitro* cytotoxicity measurements

The biocompatibility of the colloidal suspension was determined using a CellTiter-Glo luminescent cell viability assay (Promega), where intracellular adenosine triphosphate (ATP) was quantified using a luminometer (Victor 3, Perkin-Elmer). Human prostate cancer (DU145) and normal mouse hepatocyte (NCTC1469) cell lines (both cell lines were purchased from American Type Culture Collection, Rockville, MD, USA) were seeded on a 24-well cell culture plate and incubated for 24 h (5 vol% CO_2_, 37 °C). Five test solutions (1.6, 7.9, 15.7, 31.4, and 78.6 μg Gd per mL) were prepared *via* dilution of the concentrated colloidal suspension with a sterile phosphate-buffered saline solution, and ∼2 μL aliquots were used to treat the cells, which were subsequently incubated for 48 h. The viability of the treated cells was measured and normalized with respect to that of untreated control cells. Each measurement was performed in duplicate to obtain the average cell viabilities.

### Animal experiment

This study was performed in accordance with the Korean guidelines and approved by the animal research committee of Kyungpook National University.

### *In vivo T*_1_ MR image measurements in the mouse

One SD (Sparague Dawley®) mouse was used for *in vivo* test. *In vivo T*_1_ MR images were acquired using the same MRI scanner that was used for the water proton relaxation time measurements. For imaging, the mouse (∼120 g) was anesthetized using 1.5% isoflurane in oxygen. Measurements were performed before and after injection of the colloidal suspension into the mouse tail vein. The injection dose was typically ∼0.05 mmol Gd per kg. After the measurement, the mouse was revived from the anesthesia and placed in a cage with free access to food and water. During the measurement, the temperature of the mouse was maintained at ∼37 °C using a warm water blanket. The parameters used for the measurements were as follows: *H* = 1.5 T; *T* = 37 °C; NEX = 4; FOV = 9 mm; phase FOV = 0.5; matrix size = 256 × 192; slice thickness = 1 mm; spacing gap = 0.5 mm; pixel bandwidth = 15.63 Hz; TR = 500 ms; and TE = 13 ms.

## Results and discussion

### Particle diameter, hydrodynamic diameter, and crystal structure

**Fig. 2:**
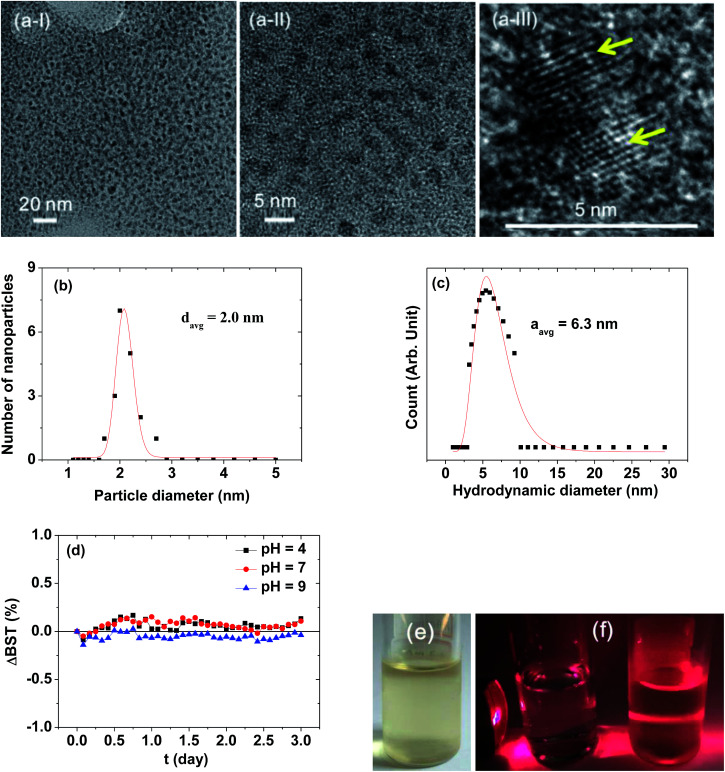
(a) HRTEM images at different magnifications [arrows in (a-III) indicate ultrasmall Gd_2_O_3_ nanoparticle colloids], (b) a log normal function fit to the observed diameter distribution, (c) a log normal function fit to the observed hydrodynamic diameter distribution, (d) plots of ΔBST as a function of time, (e) an aqueous sample solution photo showing transparency and excellent colloidal stability, and (f) the Tyndall effect showing light scattering caused by colloids: the left vial contains triple distilled water and the right vial contains a sample solution (a commercial laser point was used as a light source).

Fig. 2a presents HRTEM images, showing a monodisperse particle size distribution. The average particle diameter (*d*_avg_) was estimated to be 2.0 ± 0.1 nm from a log-normal function fit to the observed particle diameter distribution (Fig. 2b and Table 1). The average nanoparticle hydrodynamic diameter (*a*_avg_) was estimated to be 6.3 ± 0.1 nm from a log-normal function fit to the observed hydrodynamic diameter distribution (Fig. 2c and Table 1). The colloidal stability was investigated by measuring the BST of NIR beam as a function of *t* for 3 days. The ΔBST (*t*) corresponding to average BST (*t*) minus average BST (0) in which the average BST (*t*) is the average of backscattered NIR beams for all *h* (*h* = 5–10 mm) at a scan time *t*, exhibited negligible deviations from zero (Fig. 2d), confirming the stable colloidal suspensions at solution pH = 4–9. Note that the ΔBST (*t*) is zero for ideal colloids. A sample solution photo is provided in Fig. 2e. The colloidal suspension was transparent and did not settle at the bottom of the vial for at least six months, indicating excellent colloidal stability. The Tyndall effect supported the presence of colloids (Fig. 2f): the right vial containing a sample solution exhibited light scattering due to the colloidal suspension, whereas the left vial containing triple distilled water did not.

**Table 1:** Properties of the ultrasmall Gd_2_O_3_ nanoparticle colloid synthesized in this study^a^

*d*_avg_ (nm)	*a*_avg_ (nm)	PAA surface-coating amount			Water proton relaxivities (22 °C, 1.5 T)	
		*P* (weight%)	*σ* (nm^−2^)	*N*_NP_ (polymers)	*r*_1_ (s^−1^ mM^−1^)	*r*_2_ (s^−1^ mM^−1^)
2.0 ± 0.1	6.3 ± 0.1	53.7 ± 0.5	1.0 ± 0.1	13 ± 1	31.0 ± 0.1	37.4 ± 0.1

a *P*: average weight percent of PAA coated per nanoparticle. *σ*: grafting density corresponding to the number of PAA coated per nanoparticle unit surface area. *N*_NP_: number of PAA coated per nanoparticle.

**Fig. 3:**
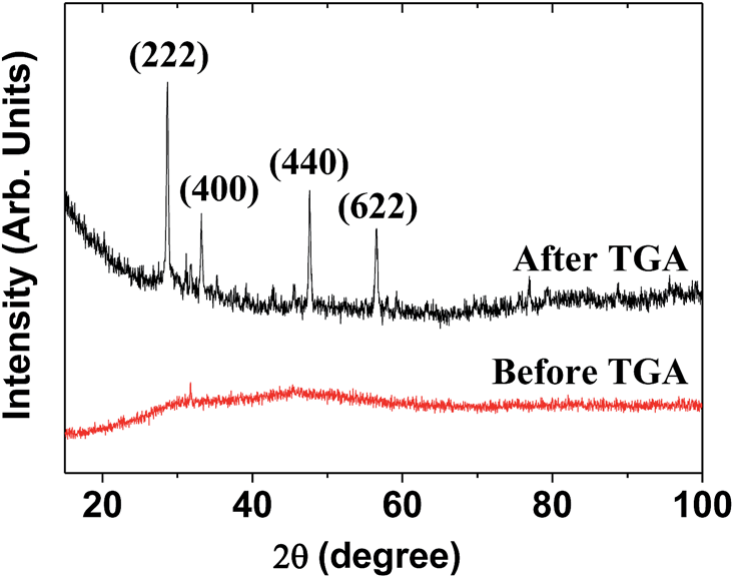
XRD patterns before and after TGA. The strong peaks in the TGA-treated sample were assigned with (*hkl*) Miller indices for cubic Gd_2_O_3_.

The XRD pattern of the as-prepared powder sample was broad and amorphous (the bottom XRD pattern in Fig. 3) owing to the ultrasmall particle size. However, after TGA, the sample exhibited the cubic structure of bulk Gd_2_O_3_ (the top XRD pattern in Fig. 3), which is attributed to crystal growth during exposure to temperatures up to 900 °C, as previously observed.^28^ The estimated lattice constant of the TGA-treated powder sample was 10.815 Å, which agrees with the reported value of 10.813 Å.^29^

### Surface-coating results

**Fig. 4:**
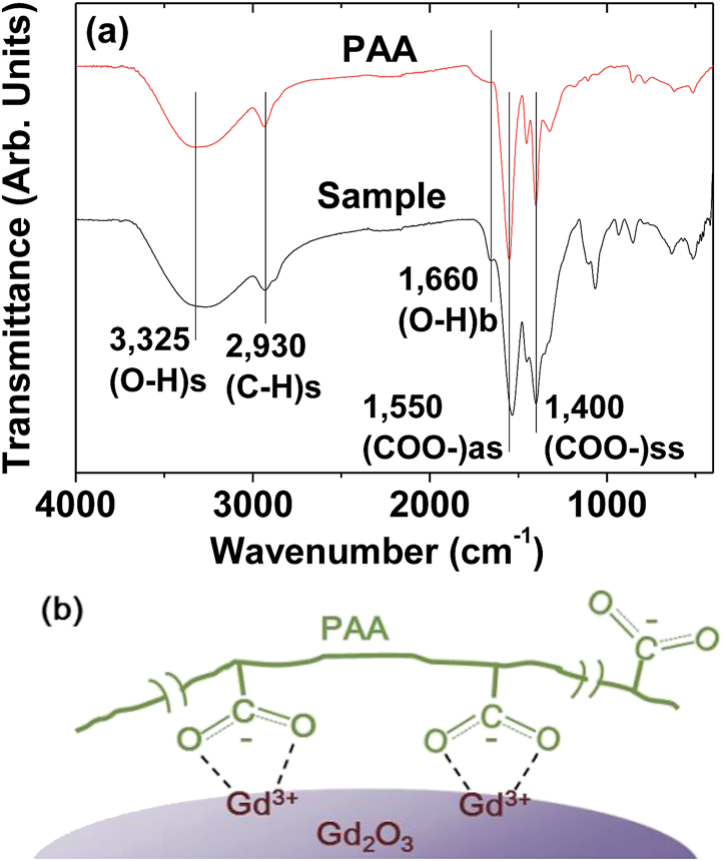
(a) FT-IR absorption spectra of a powder sample and free PAA (*M*_w_ = ∼5100 Da): 3325 cm^−1^ (O–H stretch from water); 2930 cm^−1^ (C–H stretch from PAA); 1660 cm^−1^ (O–H bend from water); 1550 cm^−1^ (COO^−^ antisymmetric stretch from PAA); and 1400 cm^−1^ (COO^−^ symmetric stretch from PAA). (b) Surface-coating structure of PAA on the ultrasmall Gd_2_O_3_ nanoparticle surface.

**Fig. 5:**
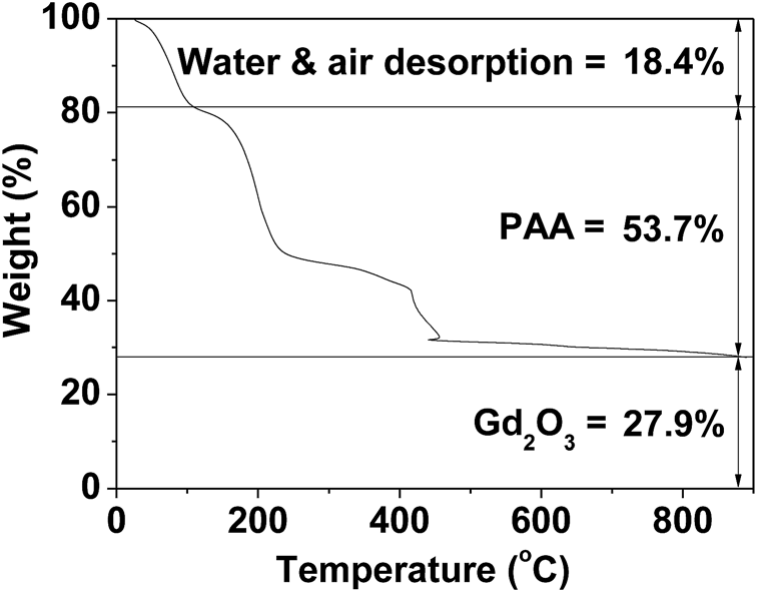
TGA curve showing the PAA surface-coating amount (53.7%) in weight percent. The weight percents of water and air (18.4%) and Gd_2_O_3_ (27.9%) are also provided.

The PAA-coating on the nanoparticle surface was investigated by recording the FT-IR absorption spectrum. The FT-IR absorption spectrum of free PAA was also recorded for comparison (the top FT-IR absorption spectrum in Fig. 4a). The sample featured characteristic PAA vibrations at 2930 cm^−1^ (C–H stretching), 1550 cm^−1^ (COO^−^ antisymmetric stretching), and 1400 cm^−1^ (COO^−^ symmetric stretching) (the bottom FT-IR absorption spectrum in Fig. 4a). PAA binds to the nanoparticle through electrostatic bonding between the COO^−^ groups of PAA and Gd^3+^ ions exposed on the nanoparticle surface. This bonding corresponds to hard acid (COO^−^ group of PAA) – hard base (Gd^3+^ ion exposed on the nanoparticle surface) type of bonding.^30–32^ Because each PAA (*M*_w_ = ∼5100 Da) has ∼54 monomer units and each monomer has a COO^−^ group (Fig. 1), multiple bonding between many COO^−^ groups of PAA and the nanoparticle is possible, forming a stable nanoparticle colloid. According to this, the surface-coating structure of PAA on the ultrasmall Gd_2_O_3_ nanoparticle surface is drawn in Fig. 4b. As estimated from TGA data (Fig. 5), approximately 13 PAA polymers were coated on each nanoparticle surface. Among approximately 54 COO^−^ groups per PAA, part of them are conjugated to Gd^3+^ ions exposed on the nanoparticle surface and the remaining ones are free.

The amount (*P*) of surface-coated PAA in weight percent was estimated to be 53.7 ± 0.5% by measuring the mass loss in a TGA curve, after considering the water and air desorption (18.4 ± 0.5%) between room temperature and ∼105 °C (Fig. 5 and Table 1). The remaining mass was due to Gd_2_O_3_ (27.9 ± 0.5%), which was consistent with 31.0% estimated using the Gd weight percent of 26.9 obtained from ICPAES analysis of the powder sample. The grafting density (*σ*), which corresponds to the average number of PAA coated per nanoparticle unit surface area,^33^ was estimated to be 1.0 ± 0.1 nm^−2^ using the bulk density of Gd_2_O_3_ (7.41 g cm^−3^),^34^ the aforementioned estimated value of *P*, and the average particle diameter determined *via* HRTEM imaging. By multiplying *σ* by the nanoparticle surface area (π*d*_avg_^2^), the average number (*N*_NP_) of PAA coated per nanoparticle was estimated to be 13 ± 1 (Table 1). This large value indicates that each ultrasmall Gd_2_O_3_ nanoparticle was sufficiently coated with PAA. This explains the excellent colloidal stability and biocompatibility that were observed in this study.

### Water proton relaxivities and map images

**Fig. 6:**
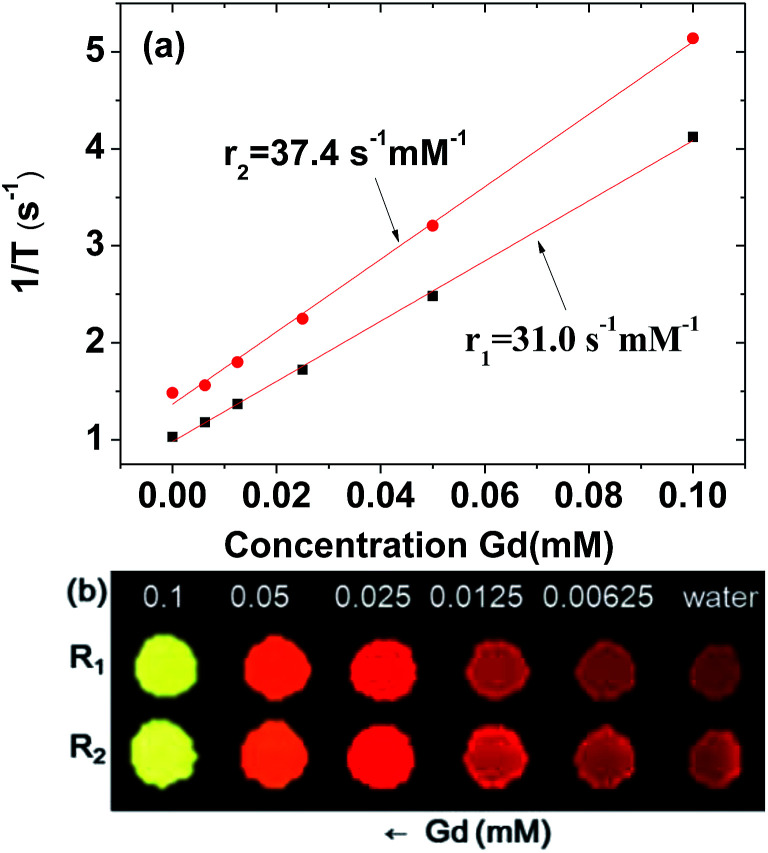
(a) Plots of 1/*T*_1_ and 1/*T*_2_ as a function of the Gd concentration (the slopes correspond to the *r*_1_ and *r*_2_ values, respectively). (b) *R*_1_ and *R*_2_ map images showing the dose-dependent contrast enhancements.

To estimate *r*_1_ and *r*_2_ values, inverse water proton relaxation times (1/*T*_1_ and 1/*T*_2_) were plotted as a function of the Gd concentration (Fig. 6a). The *r*_1_ and *r*_2_ values were estimated to be 31.0 ± 0.1 and 37.4 ± 0.1 s^−1^ mM^−1^ (*r*_2_/*r*_1_ = 1.2), respectively, from the corresponding slopes (Table 1). This high *r*_1_ value and *r*_2_/*r*_1_ ratio close to one, suggest that the synthesized ultrasmall Gd_2_O_3_ nanoparticle colloids are suitable for high-performance *T*_1_ MRI-CA. This is confirmed *in vitro* by their *R*_1_ and *R*_2_ map images, which show clear dose-dependent contrast enhancements (Fig. 6b).

The *r*_1_ value of the ultrasmall Gd_2_O_3_ nanoparticle colloid was compared with those of other Gd-nanosystems (Table 2). First of all, it was ∼8 times higher than those^4–6^ of commercial Gd-chelates. It was also higher than those of Gd_2_O_3_ nanoparticles with larger particle diameters,^15,35,36^ primarily owing to its higher surface-to-volume ratio. For the similar particle size, the nanoplate^14^ exhibited an *r*_1_ value of 47.2 s^−1^ mM^−1^ which is higher than that of the ultrasmall Gd_2_O_3_ nanoparticle colloid. This is likely because the surface area of the nanoplate is larger than that of the spherical nanoparticle. Our previous study indicated that the optimal particle diameter of Gd_2_O_3_ nanoparticles for an optimal *r*_1_ value is approximately 2 nm.^23^ A similar particle diameter dependence of the *r*_1_ value was observed in gadolinium oxide nanoplates,^14^ hybrid gadolinium oxide nanoparticles,^20^ and ultrasmall NaGdF_4_ nanoparticles.^37^ This implies that the most suitable particle diameter of Gd_2_O_3_ nanoparticles for high-performance *T*_1_ MRI-CAs is approximately 2 nm, which was used in this study.

**Table 2:** *r*_1_ and *r*_2_/*r*_1_ values for various Gd_2_O_3_ nanosystems, a free Gd^3+^ ion, and Gd-DTPA^a^

Chemical	Particle diameter (nm)	Ligand	Temperature (°C)	Applied field (*T*)	*r*_1_ (s^−1^ mM^−1^)	*r*_2_/*r*_1_	Ref.
Gd^3+^	—	DTPA	19.5	1.5	4.1	1.1	6
Free Gd^3+^	—	—	19.5	1.5	10.5	1.2	6
Gd_2_O_3_ nanoplate	2	Oleic acid	—	1.41	8.0	3.3	14
Gd_2_O_3_ nanoplate	2	PAA–OA	—	1.41	47.2	1.7	14
Gd_2_O_3_ nanoparticle	3–5	Mal-PEG-NHS-MPTS	22	1.5	22.8	1.4	15
Gd_2_O_3_ nanoparticle	20–40	Dextran	37	7.05	4.8	3.5	35
Gd_2_O_3_ nanoparticle	2.0	PAA	22	1.5	31.0	1.2	This work

a DTPA: diethyleneaminepentaacetic acid. Mal-PEG-NHS-MPTS: α-maleinimido-ω-carboxysuccinimidyl ester poly(ethylene glycol)-(3-mercaptopropyl)tri-methoxysilane. PAA–OA: polyacrylic acid–octylamine.

### Cooperative induction model for the observed high *r*_1_ value

**Fig. 7:**
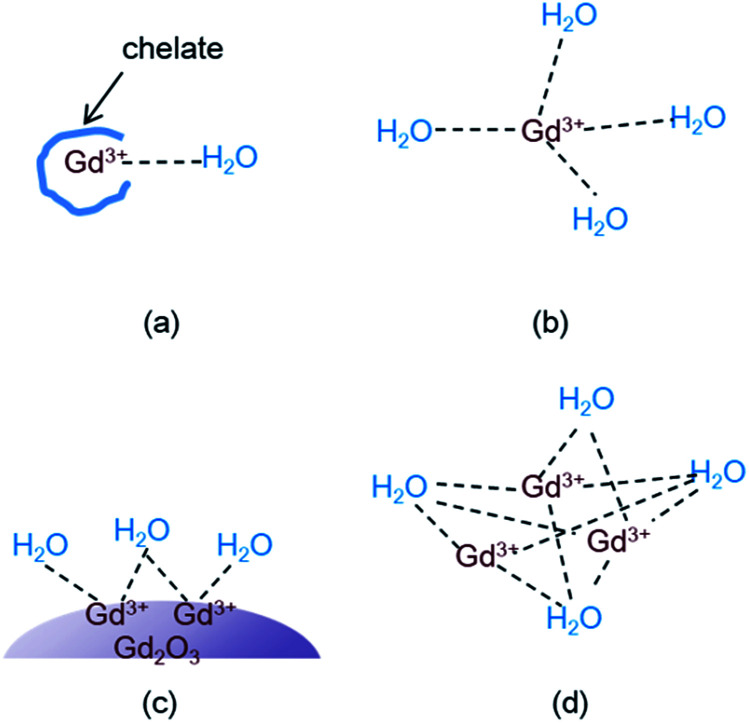
Four Gd-systems showing the interaction (labelled as dotted lines) between Gd^3+^ ions and water molecules: (a) Gd^3+^-chelate (the chelate is drawn arbitrarily), (b) free Gd^3+^ ion, (c) ultrasmall Gd_2_O_3_ nanoparticle, and (d) dense Gd^3+^ ion cluster.

Theory of water proton relaxation is very complex.^4,5,38^ To understand high *r*_1_ values observed in this study and in various Gd-nanosystems,^14,24–26^ however, a simple cooperative induction model was empirically proposed here. In this model, several Gd^3+^ ions on the nanoparticle surface or in the dense Gd^3+^ ion cluster cooperatively induce the longitudinal water proton relaxation of a water molecule. Therefore, the *r*_1_ value increases with increasing the number (*N*) of the Gd^3+^ ions interacting with a water molecule because a water molecule experiences stronger induction of its longitudinal water proton relaxation if many Gd^3+^ ions cooperatively induce its longitudinal water proton relaxation than by a single Gd^3+^ ion. In addition the *r*_1_ value increases with increasing the coordination number (*q*) of the Gd^3+^ ion with water molecules. Therefore, as the sum of the values (*N* + *q*) increases, the *r*_1_ value increases. However, the *N* (*i.e.* the cooperative induction) effect on *r*_1_ is more significant than the *q* effect as described below. To illustrate this and the cooperative induction, four Gd-systems are depicted in Fig. 7. Both the Gd-chelate and free Gd^3+^ ion have no cooperative induction effect because their *N* equals 1. Therefore, as given in Table 2, the higher *r*_1_ value of the free Gd^3+^ ion (1 ≤ *q* ≤ 9) compared with the Gd-chelate (*q* = 1) is owing to its higher *q* value. This study revealed that *r*_1_ value of the ultrasmall Gd_2_O_3_ nanoparticle (1 ≤ *q* ≤ 6 for a cubic Gd_2_O_3_, *N* > 1) was ∼3 times higher than that of the free Gd^3+^ ion even though the latter has a higher *q* value. Therefore, the higher *r*_1_ value of the former is due to its higher *N* value compared with the latter. This indicates that the *N* should play a more significant role in *r*_1_ value than the *q* does. Both *N* and *q* values of the dense Gd^3+^ ion cluster are higher than the respective values of the ultrasmall Gd_2_O_3_ nanoparticle: *q* of the Gd^3+^ ion in the dense Gd^3+^ ion cluster is similar to that of the free Gd^3+^ ion and thus higher than that of the nanoparticle, and *N* of the Gd^3+^ ion cluster is also higher than that of the nanoparticle because all the Gd^3+^ ions in the Gd^3+^ ion clusters can contribute to inducing the longitudinal water proton relaxation whereas in nanoparticles, only the Gd^3+^ ions exposed on the nanoparticle surface dominantly contribute to the induction. This explains *r*_1_ values of 70–173 s^−1^ mM^−1^ of the dense Gd^3+^ ion clusters prepared inside and outside CNTs,^24–26^ which were 2 to 6 times higher than that of the ultrasmall Gd_2_O_3_ nanoparticle in this study. In this way, all the experimental observations of *r*_1_ (Gd^3+^ ion cluster) > *r*_1_ (ultrasmall Gd_2_O_3_ nanoparticle) > *r*_1_ (free Gd^3+^) > *r*_1_ (Gd^3+^-chelate) can be explained using this simple model. Therefore, the cooperative induction effect plays an important role in *r*_1_ value and thus should be considered in designing high-performance *T*_1_ MRI-CAs with high *r*_1_ values.

### Cytotoxicity results

**Fig. 8:**
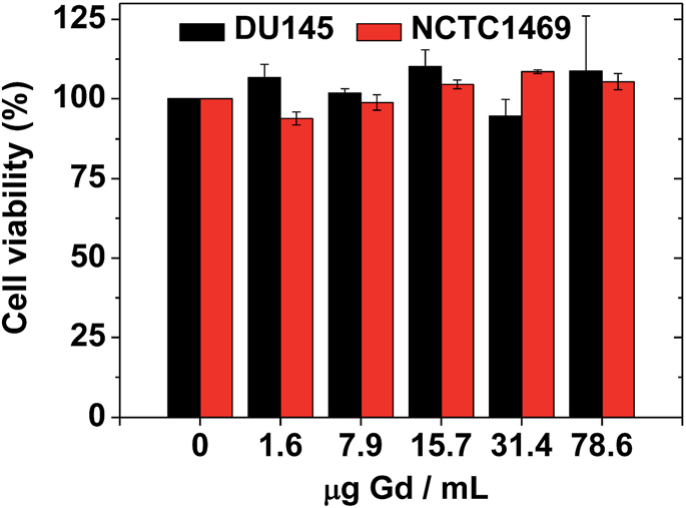
*In vitro* cellular cytotoxicities of a sample solution in DU145 and NCTC1469 cell lines, showing non-toxicity up to 78.6 μg Gd per mL.

The biocompatibility of the colloidal suspension was demonstrated *in vitro* by measuring cellular cytotoxicities using DU145 and NCTC1469 cell lines. As shown in Fig. 8, the colloidal suspension was non-toxic up to 78.6 μg Gd per mL and thus, suitable for *in vivo* applications. Recently, accumulation of Gd in the brain has been a big issue due to possible neurotoxicity. Therefore, a further study to evaluate the Gd deposition in the brain *ex vivo* is needed, which will be carried out in a future.

### *In vivo T*_1_ MR images

**Fig. 9:**
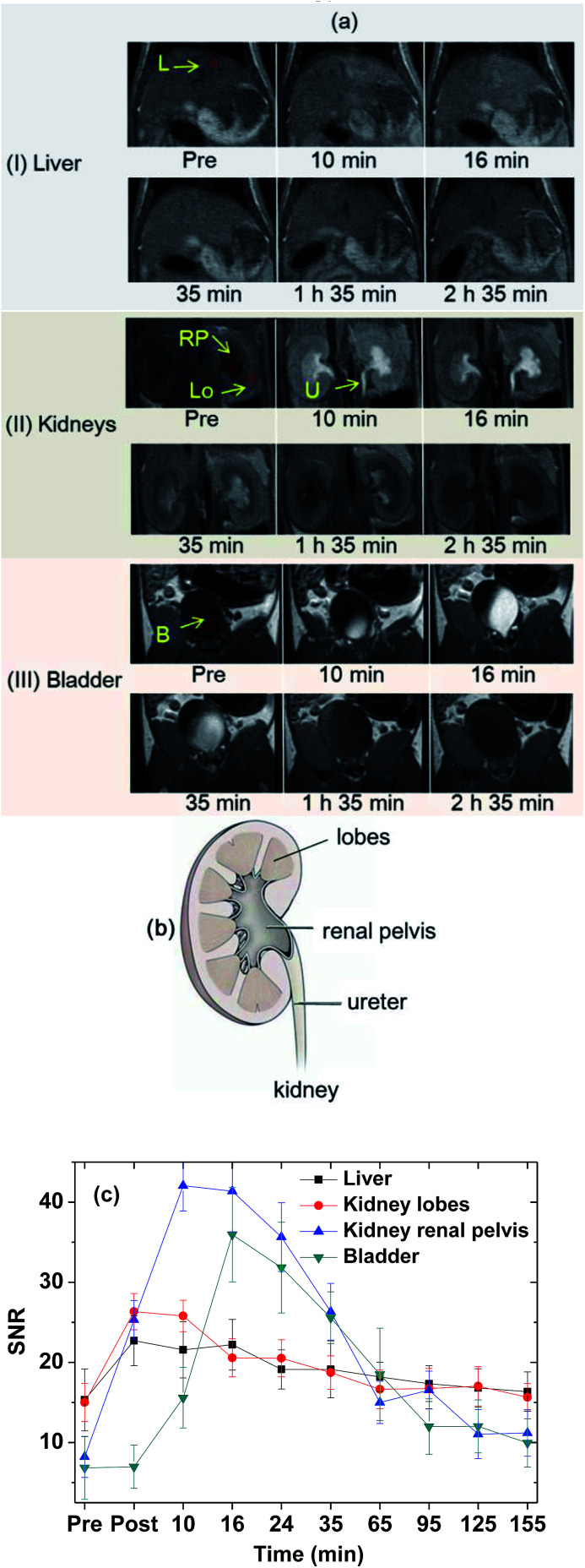
(a) *In vivo T*_1_ MR images in various organs of a mouse (circles label ROIs used for SNR plots) after intravenous administration: (I) liver (labelled as “L”), (II) kidneys (“Lo”: kidney lobes, “RP”: kidney renal pelvis, and “U”: ureter), and (III) bladder (labelled as “B”), (b) nomenclature of the lobes, renal pelvis, and ureter in the kidneys, and (c) SNR plots of ROIs in various organs of the mouse as a function of time (pre = before and post = just after intravenous administration).

The effectiveness of the colloidal suspension as a high-performance *T*_1_ MRI contrast agent was evaluated by taking *in vivo T*_1_ MR images in a mouse after intravenous administration into the tail. As shown in Fig. 9a, positive contrast enhancements were observed in the liver, kidney lobes, kidney renal pelvis, ureter, and bladder and then decreased with time because of the excretion of the nanoparticle colloids through the bladder. In addition, owing to the high contrasts and ultrasmall diameters, excretion of the nanoparticle colloids through the lobes, renal pelvis, and ureter of the kidneys (see Fig. 9b for nomenclature) was observed. To clearly show contrast changes with time, the signal-to-noise ratios (SNRs) of region of interests (ROIs) in organs (labelled as circles in Fig. 9a) were plotted as a function of time (Fig. 9c), exhibiting that the contrasts initially increased, reached the maxima, and then decreased with time in all organs. For the liver, the nanoparticle colloids showed the highest contrast enhancement around 10 min after intravenous administration and then the contrast gradually decreased with time. This contrast enhancement pattern in the liver suggests that the nanoparticle colloids were not taken up by reticuloendothelial system of the liver. The colloidal suspension was finally excreted through the bladder, which is consistent with the other ultrasmall nanoparticle systems' behavior with *d* < 3 nm.^20–22^

As demonstrated, the ultrasmall Gd_2_O_3_ nanoparticle colloids were stable and non-toxic and excreted through the renal system owing to their ultrasmall particle diameter, proving their suitability for use as *T*_1_ MRI-CA. In addition they exhibited a very high *r*_1_ value with an *r*_2_/*r*_1_ ratio close to one, and as a result, high contrast *T*_1_ MR images in the mouse. Therefore, the ultrasmall Gd_2_O_3_ nanoparticle colloids synthesized in this study can be used as a high-performance *T*_1_ MRI-CA.

## Conclusions

We reported the facile one-pot synthesis and characterization of ultrasmall Gd_2_O_3_ nanoparticle colloids (coating material = PAA, *M*_w_ = ∼5100 Da) *in vitro* and *in vivo*. The results are as follows.

(1) The particle diameter was monodisperse and ultrasmall (core *d*_avg_ = 2.0 ± 0.1 nm).

(2) The colloidal suspension was stable and biocompatible owing to the PAA-coating on the nanoparticle surface. Cytotoxicity tests using two cell lines showed non-toxicity up to 78.6 μg Gd per mL.

(3) The colloidal suspension exhibited *r*_1_ = 31.0 ± 0.1 and *r*_2_ = 37.4 ± 0.1 s^−1^ mM^−1^ (*r*_2_/*r*_1_ = 1.2). The *r*_1_ value was ∼8 times higher than those of commercial Gd-chelates. We attribute this to the ultrasmall particle diameter and the hydrophilic PAA-coating on the nanoparticle surface. The cooperative induction model was proposed to explain this high *r*_1_ value.

(4) The colloidal suspension exhibited high contrast *T*_1_ MR images in various organs of the mouse after intravenous administration and was finally excreted through the renal system. Therefore, the synthesized ultrasmall Gd_2_O_3_ nanoparticle colloids should be a potential candidate for use as a high-performance *T*_1_ MRI-CA.

## Author contributions

Xu Miao carried out synthesis, measurements and characterizations, Son Long Ho contributed to the synthesis, Hyunsil Cha and Yongmin Chang measured relaxivities and MR images, In Taek Oh and Kwon Seok Chae measured cellular toxicities, Tirusew Tegafaw, Ahmad Mohammad Yaseen, Shanti Marasini, Adibehalsadat Ghazanfari and Huan Yue contributed to experiments, and Gang Ho Lee wrote the manuscript.

## Conflicts of interest

There are no conflicts to declare.

## Supplementary Material
